# Clinical Outcomes of Repeated Sodium Polynucleotide Injections in Knee Osteoarthritis: Large-Scale, Retrospective Cohort Study

**DOI:** 10.3390/jcm14238358

**Published:** 2025-11-25

**Authors:** Jiyon Bok, Bongseong Kim, Kyungdo Han, Dongwook Shin, Hasuk Bae

**Affiliations:** 1 Department of Physical Medicine and Rehabilitation, Ewha Womans University College of Medicine, Mokdong Hospital, Seoul 07985, Republic of Korea; acebhs@gmail.com; 2 Department of Statistics and Actuarial Science, Soongsil University, Seoul 06978, Republic of Korea; qhdtjd12@gmail.com (B.K.); hkd917@naver.com (K.H.); 3 Department of Family Medicine & Supportive Care Center, Samsung Medical Center, Sungkyunkwan University School of Medicine, Seoul 06351, Republic of Korea; dwshin.md@gmail.com; 4 Department of Clinical Research Design and Evaluation, Samsung Advanced Institute for Health Science and Technology (SAIHST), Sungkyunkwan University, Seoul 06351, Republic of Korea

**Keywords:** knee osteoarthritis, sodium polynucleotide, intra-articular injection, viscosupplementation, surgery delay, repeated treatment, real-world evidence, retrospective cohort study

## Abstract

**Background/Objectives**: Sodium polynucleotide (PN) injection has recently been considered as a potential intra-articular therapy for knee osteoarthritis (OA); however, there is limited evidence regarding the long-term consistency of repeated PN cycles. To evaluate the clinical effectiveness of repeated intra-articular PN injections after the initial 6 months of therapy in patients with knee OA, using nationwide claims data. **Methods**: We conducted a retrospective cohort study using data from the Korea Health Insurance Review and Assessment Service collected between 2020 and 2023. Patients who received PN injections for knee OA were classified into two groups based on the treatment cycle: Group 1 (single cycle) and Group 2 (re-administration). Surgical outcomes and symptomatic indicators, including pain-related hospital visits, arthrocentesis, nonsteroidal anti-inflammatory drug prescriptions, and antidepressant prescriptions, were analyzed. **Results**: A total of 142,322 patients were included in this study. Readministration of PN was associated with significantly lower rates of total knee arthroplasty (2.31% vs. 4.92%, *p* < 0.0001) and delayed time to surgery (252.0 vs. 176.6 days, *p* < 0.0001). Similar trends were observed for hemiarthroplasty, with a lower incidence (0.28% vs. 0.55%, *p* < 0.0001) and longer time to surgery (240.7 vs. 162.2 days, *p* < 0.0001) in the readministration group. All groups showed a timewise reduction in pain-related hospital visits and instances of arthrocentesis. Safety outcomes were not assessed in this claim-based dataset. **Conclusions**: Repeated cycles of PN injections provide sustained clinical benefits and may effectively delay the need for surgical intervention in patients with knee OA, supporting their possible role as a long-term conservative treatment option. Radiographic severity and safety outcomes were unavailable in this claims dataset, limiting the adjustment for baseline OA severity and restricting causal interpretation.

## 1. Introduction

Osteoarthritis (OA) is a common disease and leading cause of disability among older adults. Knee OA is a substantial burden, marked by its chronicity and high incidence. In Korea, a large-scale population-based study reported that among Korean community residents aged 50 years and older, the prevalence of radiographic knee OA was 37.3% and that of symptomatic knee OA was 24.2%, with significantly higher rates observed in women and those with obesity or low education levels [1]. OA imposes a substantial and growing economic burden owing to its high prevalence, treatment costs, disability, and work-related losses, making it a worldwide concern [2].

OA is increasingly recognized as a multifactorial disease influenced by a broad range of biomechanical, metabolic, and environmental factors. Regarding age-related degeneration, key contributors include previous joint injuries, occupational mechanical load, high-impact sports activity, obesity, and sex-related biological differences, all of which shape the trajectory of cartilage deterioration and symptom progression. Recent biomechanical studies have highlighted that altered cartilage stress distribution, impaired shock absorption, and repetitive shear loading play central roles in accelerating the structural damage in knee OA [3]. These findings emphasize the importance of early and objective assessment of joint mechanics to prevent structural progression and guide individualized treatment strategies. Incorporating such multifactorial influences provides an essential clinical and epidemiological context when evaluating non-surgical interventions such as intra-articular (IA) injection therapy.

IA injection therapy is widely used to treat knee OA, particularly in patients who are not candidates for surgery and require conservative treatment. Hyaluronic acid (HA) has traditionally been used as a viscosupplement. However, recent evidence has questioned its therapeutic benefits. The 2021 clinical guidelines from the American Academy of Orthopaedic Surgeons (AAOS) advise against the routine use of intra-articular hyaluronic acid (IA HA) injections because of inconsistent clinical benefits [4]. Similarly, the 2019 guidelines of the American College of Rheumatology and Arthritis Foundation (ACR/AF) conditionally recommend IA HA for knee OA [5]. In contrast, the Korean Knee Society continues to support the use of IA HA as an effective treatment option, highlighting the regional differences in clinical practice and patient response [6].

With growing debate over the efficacy of HA, sodium polynucleotide (PN), a biopolymer derived from highly purified DNA, has gained attention as an alternative to IA. PN products act through physical restoration mechanisms by binding to water molecules to form a three-dimensional viscoelastic gel that reduces mechanical friction in the joint area and provides symptom relief without pharmacological action [7,8,9]. Several clinical studies demonstrated the efficacy of PN in patients with knee OA. In a randomized controlled trial, Vanelli et al. reported significant improvements in knee function and pain after PN injections [7]. A randomized, double-blind clinical study showed that IA injection of PN with HA resulted in superior improvement in pain and knee function compared with HA alone in patients with knee OA [8]. In addition, a 2-year randomized, double-blind trial demonstrated that IA PN combined with HA provided greater and longer-lasting pain relief and functional improvement than HA alone in patients with knee OA [9]. In this pilot study, the intra-articular injection of polynucleotide sodium resulted in significant improvement in pain and function in patients with knee osteoarthritis, and its efficacy was comparable to that of sodium hyaluronate and cross-linked sodium hyaluronate [10].

Although PN has shown promising clinical effects, the intra-articular behavior of this biopolymer is not yet fully understood. Unlike hyaluronic acid, whose intra-articular residence time and degradation kinetics have been partly described, PN-specific pharmacokinetic characteristics remain largely unreported. PN is a highly purified DNA-based biopolymer [7,9], and as with other DNA polymers, it is theoretically expected to undergo enzymatic degradation by nucleases into smaller nucleotide fragments; however, quantitative data on the intra-articular residence time or degradation rates are not currently available. This knowledge gap underscores the need for large-scale real-world studies to evaluate whether the therapeutic benefits are consistently maintained with repeated PN injections.

Recently, Kim et al. conducted a multicenter retrospective study in Korea to evaluate the clinical effectiveness and safety of repeated IA PN (Conjuran^Ⓡ^) injections. The authors suggested that PN injections could be safely repeated at six-month intervals while maintaining clinical benefits, with no significant adverse events (AE) observed during repeated treatment cycles [11]. As one of the few studies focusing on Korean patients, this study provides important regional insights into the potential of repeat PN. Lee et al. described a case of intractable knee OA that showed marked symptomatic improvement without safety concerns after multiple PN injections [12].

However, because PN has only recently been used clinically, there is still a lack of large-scale real-world evidence to assess the clinical consistency of repeated PN injection cycles for long-term effects. No prior study has systematically compared the outcomes of first and subsequent PN treatments using national-level data. Therefore, this study aimed to evaluate whether repeated IA PN injections provide sustained therapeutic benefits using a nationwide retrospective cohort derived from the Korean national health data.

## 2. Materials and Methods

### 2.1. Data Collection

Data were collected from patients with knee OA who received IA PN injections between 1 January 2020 and 30 September 2023. This study was approved by the Public Institutional Review Board (SMC IRB 2024-05-111). Data were obtained from the Korean Health Insurance Review and Assessment Service and personal identifiers were removed.

The Health Insurance Review and Assessment Service data provides information on the overall number of claims, age, sex, insurance type (Health Insurance/Medical Aid), healthcare facility type (tertiary hospitals, general hospitals, clinics, long-term care hospitals, etc.), facility location, medical departments, diagnoses, and treatment details. Specifically, for PN injections and OA, the data included history of OA, injection frequency, injection dates, comorbidities, medication subscriptions, and concomitant surgery. However, the HIRA claims database does not contain imaging results (e.g., Kellgren–Lawrence grades), pain scales, or functional outcome measures because these data were not collected for administrative purposes. Therefore, radiographic OA severity and patient-reported outcomes were not analyzed. Medication adherence could not be assessed because the claims data capture only prescription issuance rather than actual medication intake.

Potential missing values, such as age, sex, and insurance type, were examined for variables that were commonly incomplete in the administrative claims data. In this dataset, all included patients had complete information on these variables, and no observations required exclusion owing to missing data. Duplicate or conflicting claims were addressed by consolidating multiple claims recorded on the same date into a single clinical encounter. When two or more pain-related visits appeared in the database on the same calendar day, they were counted as one visit to avoid artificial inflation of the visit frequency.

### 2.2. Patient Selection

Based on data reported to the National Health Insurance Service up to 30 September 2023, patients were analyzed as follows:

Exposure to IA PN therapy was identified using the Korean treatment material code M2094, which includes three approved PN injection products. All claims with codes under the M2094 prefix were used to identify PN injection cases:

- M2094158: Conjuran^®^ (PharmaResearch);
- M2094058: Condrotide (manufactured by Mastelli, Licensed by Pharma Research, Korea).

Patients who were administered any of these products were included in the analysis if they satisfied other eligibility criteria. M2094011: Vitaran^®^ (BR Pharm) also falls under M2094 codes, but notably, Vitaran, launched in April 2022, was excluded from the analysis, as the study population was defined to include only patients who had received injections by the fourth quarter of 2021, to allow for a one-year follow-up period.

From an initial dataset of 807,609 patients who received IA PN (Conjuran^Ⓡ^ and Condrotide^®^) injections between 2020 and the third quarter of 2023, a total of 249,650 patients were selected for analysis after excluding those whose first injection occurred in 2022 or later. This criterion was applied to ensure a minimum one-year follow-up period following the initial administration and to reflect the treatment cycle of PN, which spans six months per cycle.

Among them, 184,193 patients who completed the full first treatment cycle, defined as receiving 2–5 injections within six months, were included. Patients who received a single injection were excluded. To limit the analysis to patients with a clinically interpretable number of repeated cycles, 33,521 patients who underwent three or more treatment cycles were excluded. Patients who received three or more treatment cycles were excluded because the available follow-up after the last injection was insufficient to reliably assess long-term outcomes beyond the second cycle.

**Figure 1:**
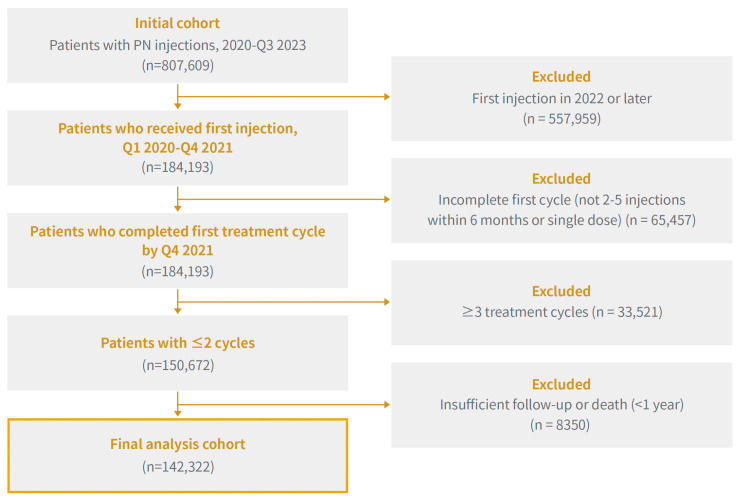
Flowchart of Patient Selection for this Study: Inclusion and Exclusion Criteria for Repeated Sodium Polynucleotide Administration in Patients with Knee Osteoarthritis (2020–2021).

Finally, 8350 patients were excluded owing to insufficient follow-up, either because they lacked a full year of observation following their last injection or died within one year of their final dose. Finally, 142,322 patients were included in the final cohort. The overall selection process is illustrated in Figure 1.

### 2.3. Definition of Treatment Cycles and Re-Administration

In this study, one cycle of IA PN injection was defined as up to five injections administered within six months of the initial injection. Readministration of injection was defined as the initiation of an additional cycle beyond the first cycle, regardless of the exact number of injections received.

Based on the number and timing of the PN injection cycles, the patients were classified into two groups, as shown in Table 1.

- Group 1 (Single-Cycle Group): patients who received only one PN injection cycle * during the study period with no subsequent re-administration.
- Group 2 (readministration group): patients who received two or more PN cycles, regardless of the interval between the first cycle and readministration.

* One cycle: up to five injections within six months from the first injection.

In Korea, the reimbursement guidelines of the Ministry of Health and Welfare (Notice No. 2021-141) allow for up to five intra-articular PN injections within six months, with each 6-month period defined as one treatment cycle. This regulatory framework provides the basis for the cycle definitions used in this study [13]. This cycle definition reflects Korean reimbursement regulations and does not originate from pharmacological or pharmacokinetic evidence. Because PN products are not uniformly approved or reimbursed worldwide and no international guidelines define a standardized injection interval, the Korean 5-injections within the 6-month rule should be interpreted as a country-specific regulatory framework rather than a universally generalizable treatment cycle.

### 2.4. Outcome

Based on these categories, two major analyses were conducted to evaluate the effect of repeat PN injections on surgical delays.

1. Primary Analysis: Group 1 (single cycle) was compared with group 2 (re-administration) to determine whether repeated PN therapy prolonged the time to surgical intervention.
2. Secondary Analysis: In addition to these main analyses, we evaluated changes in pain-related indicators, including the number of pain-related hospital visits, joint aspiration procedures, NSAID prescriptions, and antidepressant use, both within the single-cycle group (Group 1) and the re-administration group (Group 2) over time. Within-group comparisons were conducted to assess whether these indicators were significantly decreased after PN injection. Subsequently, we compared the rates of change (slopes of reduction) across the single-cycle and re-administration treatment groups to determine whether the degree of symptomatic improvement differed significantly between the groups. Pain-related hospital visits were defined as outpatient or inpatient encounters that included a diagnostic code for knee OA (M17 and M13) and were made to pain-related specialties (orthopedics, physical medicine and rehabilitation, anesthesiology and pain medicine, or neurosurgery). Visits unrelated to knee OA were excluded.

Patient-reported outcomes such as pain scores, functional assessments, or quality-of-life measures were not available in the HIRA database and therefore could not be evaluated in this study. Direct AE reporting was not available because the HIRA claims database does not include procedure-specific complication records, such as injection-site reactions, post-injection flares, or device-related events.

### 2.5. Statistical Analysis

Baseline characteristics across the three groups were compared using t-tests. Independent t-tests were used for comparisons between groups 1 and 2 As age and sex differed across the groups, adjusted means were estimated. Within-group trends over time (pre-injection, 0–6 months, and 6–12 months) were analyzed using paired *t*-tests. Changes in slope across groups were assessed using linear regression. Cox regression and propensity score–based methods were not used because the primary outcomes involved comparing event counts within fixed observation windows rather than time-to-event risks, and the key clinical covariates required for propensity modeling (e.g., radiographic severity and pain intensity) were unavailable in the claims data. Analyses were performed using SAS 9.4 (SAS Institute, Cary, NC, USA), with significance set at *p* < 0.05.

## 3. Results

### 3.1. Baseline Characteristics

A total of 142,322 patients were included in the analysis, with 115,388 in Group 1 (single cycle) and 26,934 in Group 2 (re-administration).

The mean age of the total cohort was 62.56 ± 10.58 years, with patients in Group 2 being slightly older (63.07 ± 9.65 years) compared to Groups 1 (*p* < 0.0001). The proportion of male patients differed significantly across the groups, with Group 2 showing the lowest proportion (29.77%) (*p* = 0.0004).

The number of outpatient visits to pain-related specialties (rehabilitation, orthopedics, anesthesiology, and neurosurgery) was higher in Group 2 (3.5 ± 6.04) and lower in Group 1 (3.25 ± 6.04) (*p* < 0.0001). Arthrocentesis procedures were also more frequently performed in Group 2 (0.25 ± 1.03) than in Group 1 (0.23 ± 0.97) (*p* = 0.0034). Similarly, Group 2 had a higher number of NSAID prescriptions (3.54 ± 4.09) compared to Group 1 (3.34 ± 4.08) (*p* < 0.0001).

However, the proportion of Medical Aid beneficiaries with low-income status did not differ significantly across the groups, ranging from 2.82% to 2.87% (*p* = 0.9044).

Several clinically important baseline characteristics, including body mass index, radiographic severity, pain intensity, and functional status, were not available because these data were not collected from the HIRA claims database (Table 2).

### 3.2. Primary Analysis: Surgical Outcomes

**Figure 2:**
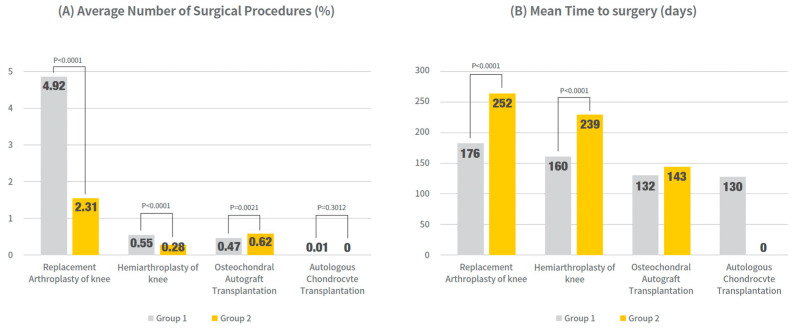
Surgical outcomes within 1 year after PN initiation: Surgery rates and timing (mean days to event) were compared between the single-cycle group (Group 1) and re-administration group (Group 2). All models were adjusted for age and sex. Values presented as mean (95% CI) and % of patients undergoing each procedure. Exact numerical values are provided in Supplementary Table S1.

For the primary analysis, the incidence and timing of knee surgeries within 1 year of PN administration were compared between the single-cycle (Group 1) and re-administration groups (Group 2), as shown in Figure 2 and Supplementary Table S1.

The rate of knee replacement arthroplasty was significantly lower in group 2 (2.31%) than in group 1 (4.92%) (*p* < 0.0001). Furthermore, the mean time to surgery was markedly longer in Group 2 (252.0 days; 95% CI, 244.4–259.6) than in Group 1 (176.6 days; 95% CI, 173.6–179.5) (*p* < 0.0001). Similar trends were observed for hemiarthroplasty, with group 2 showing a lower incidence than group 1 (0.55% vs. 0.28%, *p* < 0.0001) and delayed surgery (162.2 vs. 240.7 days, *p* < 0.0001).

For osteochondral autograft transplantation, while the incidence was slightly higher in Group 2 compared to Group 1 (0.62% vs. 0.47%, *p* = 0.0021), the mean time to surgery did not differ significantly (133.0 days; 95% CI, 124.49–141.49 vs. 143.2 days; 95% CI, 128.43–158.02) (*p* = 0.2266). Autologous chondrocyte transplantation was rare in both groups, and no significant differences were observed in frequency or timing.

### 3.3. Secondary Analysis: Pain-Related Hospital Visits, NSAID and Antidepressant Prescriptions, and Joint Aspiration Counts

**Figure 3:**
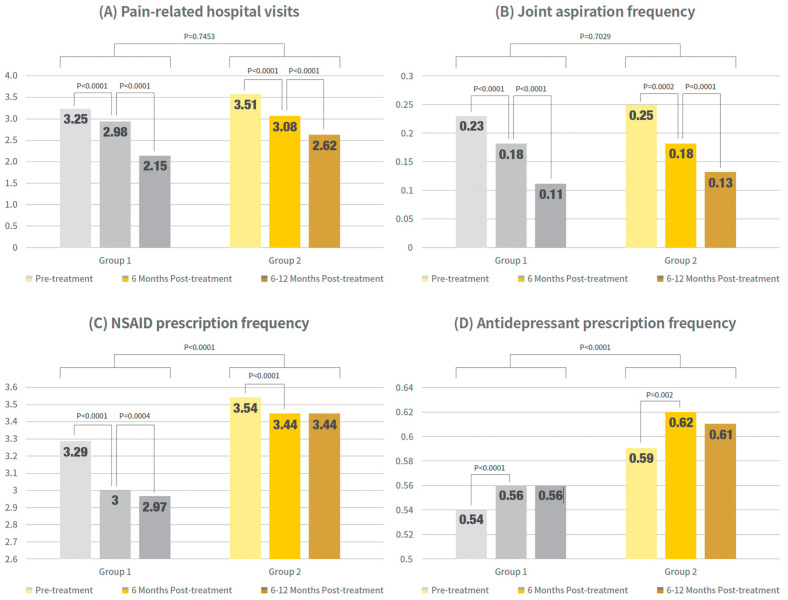
Clinical outcome trends in timewise order within groups. Bar graphs show mean values of pain-related outpatient visits, arthrocentesis procedures, NSAID prescriptions, and antidepressant prescriptions at pre-treatment, 0–6 months post-treatment, and 6–12 months post-treatment for single- and re-administration group. Exact numerical values are provided in Supplementary Tables S2–S5.

Pain-related hospital visits, NSAID and antidepressant prescriptions, and joint aspiration counts were compared within Group 1 and Group 2 in timely order after PN injection, which is shown in Figure 3 and Supplemental Tables S2–S5.

Outpatient visits to pain-related specialties and arthrocentesis procedures significantly decreased in both groups, with reductions observed from pretreatment to 6 months post-treatment and from 6 to 12 months post-treatment (*p* < 0.0001 for both the single- and re-administration groups).

In contrast, NSAID prescription patterns differed between the groups. Groups 1 and 2 showed significant reductions at 6 months compared to pretreatment (*p* < 0.0001 for both groups). However, from six months to one year, a significant reduction was observed in the single-cycle group (*p* = 0.0004), whereas no significant change was observed in the readministration group (*p* = 0.8151). In summary, NSAID prescriptions significantly decreased during the first six months in both groups, but only the single-cycle group maintained a significant declining trend for up to one year.

The prescription patterns of antidepressants were similar between groups. Both group 1 and group 2 showed a significant increase at 6 months compared to pretreatment (*p* < 0.0001 for the single-cycle group and *p* = 0.002 for the re-administration group). However, from 6 months to 1 year, no further significant increase was observed in either group (single-cycle group, *p* = 0.7547; readministration group, *p* = 0.1469).

**Figure 4:**
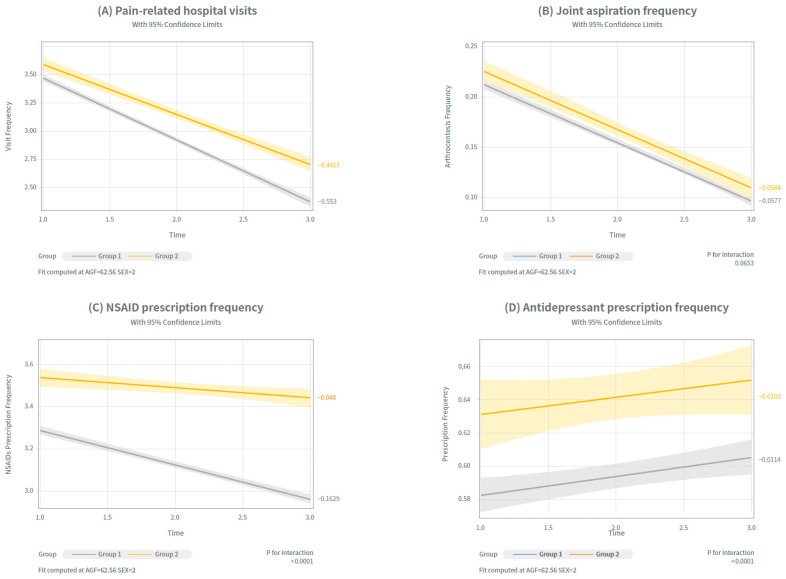
Trends in 4 clinical outcomes before and after IA PN injection across treatment groups. Trends in four clinical indicators were analyzed over time to assess symptomatic changes within each treatment group (Group 1: single-cycle; Group 2: re-administration group). Differences in slopes estimated from linear regression models between the two groups. (**A**) Pain-related hospital visits (**B**) Joint aspiration frequency (**C**) NSAID prescription frequency (**D**) Antidepressant prescription frequency. Exact numerical values are shown in Supplemental Table S6.

The rates of change (slopes of reduction) between the two groups were compared. The detailed results of these between-group slope comparisons were estimated using linear regression and are shown in Figure 4 and Supplementary Table S6.

Pain-related hospital visits showed significant differences in the slope between the groups. Group 1 exhibited steeper decline over time (adjusted mean: 3.2 → 2.9 → 2.07), compared to Group 2 (3.41 → 2.95 → 2.51). Slope comparisons revealed significant differences between the single-and re-administration groups (*p* < 0.0001), suggesting that the single cycle and re-administration groups are effective in reducing pain-related hospital visits, but have a more durable reduction in visit burden in the single-cycle group.

The number of arthrocentesis procedures decreased in both groups. Group 1 showed a reduction from 0.25 → 0.19 → 0.12 (*p* = 0.7029), and Group 2 from 0.27 → 0.19 → 0.14 (*p* = 0.7023). However, there were no statistically significant differences between the slopes. This indicated that repeated PN administration was as effective as a single-cycle treatment in reducing joint aspiration frequency.

In contrast, NSAID prescription frequency showed a markedly steeper decline in Group 1 (3.29 → 2.98 → 2.94) compared to Group 2 (3.51 → 3.39→ 3.39), with significant differences between Group 1 and Group 2 (*p* < 0.0001).

Finally, antidepressant prescription frequency did not differ significantly between the groups. Group 1 changed from 0.51 → 0.53 → 0.54 (*p* = 0.7453), and Group 2 from 0.56 → 0.59 → 0.58 (*p* = 0.7321), indicating that in both groups, prescriptions increased in timely order. The slope differences were not statistically or clinically significant (*p* = 0.7321), suggesting a limited effect of PN.

## 4. Discussion

IA PN injection has emerged as a promising intervention for knee OA. A randomized controlled trial demonstrated that at 16 weeks, the PN group exhibited a significant reduction in VAS scores, highlighting its rapid onset of action [14]. Given the growing clinical interest in viscosupplementation as a non-surgical therapeutic option for OA, previous reviews have highlighted its ability to improve joint lubrication, viscoelasticity, and shock absorption, thereby contributing to pain relief and functional improvement [15]. Furthermore, another double-blind randomized study comparing a fixed coformulation of PN and HA (PNHA) versus HA alone reported superior improvements in the WOMAC and Knee Society Scores in the PNHA group. This benefit was sustained for up to 24 months following a three-week course of injections [9].

The present study extends these findings by showing that repeated PN injections are as effective as the initial cycle in delaying surgical interventions. The analysis was restricted to patients who received one or two treatment cycles because this study’s primary aim was to assess the effectiveness of repeated PN therapy up to the second cycle. Patients who received multiple cycles were excluded, as the follow-up period after the final injection was too short to reliably evaluate long-term outcomes beyond the second cycle. However, excluding patients with ≥3 cycles—who may represent individuals with more severe or persistent symptoms—may limit the interpretability of the long-term effectiveness of PN beyond the second cycle. This limitation should be considered when interpreting the findings of this study.

Specifically, patients who underwent total or hemiknee arthroplasty showed significantly lower surgical rates and prolonged time to surgery after repeated PN administration than those who underwent a single-cycle injection. While the proportion of patients undergoing osteochondral autograft transplantation was higher in the re-administration group, the time from final injection to surgery was not significantly different from that in the single-cycle group. No statistically significant differences were observed after autologous chondrocyte transplantation, which may be attributed to the limited sample size. These findings suggest that repeated PN administration may be associated with delayed progression to surgical intervention, particularly for major procedures, such as total or partial knee arthroplasty.

When interpreting these findings, it is important to note that radiographic findings were not collected or stored in the HIRA claims system. This study did not include the Kellgren-Lawrence (K-L) grades, which are commonly used to assess OA severity. Therefore, the exact disease progression of the patients in each group could not be determined. While PN injections are clinically indicated for K-L grade 1–3 knee OA, the corresponding imaging results used to establish eligibility are unavailable in this dataset. The absence of radiographic severity measures limits our ability to adjust for baseline OA severity or evaluate whether disease progression differs across treatment cycles. Nevertheless, given that the readministration group was older, had more frequent pain-related visits and medication use, and underwent repeated PN administration, it is reasonable to infer that these patients might have had more advanced OA than those in the single-cycle group. Previous studies have also shown that older age, higher NSAID use, and increased healthcare visits are associated with greater OA severity [16]. In addition, this inference should be approached with caution, as repeated PN administration may also reflect physician or patient preferences, treatment accessibility, or non-clinical factors other than disease progression. This limitation is inherent to real-world claims data, which often lack radiographic severity measures such as the K-L grade [17]. Additionally, because the Korean healthcare system allows for frequent outpatient visits and easy access to injection-based treatments under universal coverage [18], treatment patterns, including PN utilization frequency, may differ substantially from those in other countries. Therefore, the generalizability of these findings to international populations should be interpreted with caution. However, the use of a large nationwide dataset provided robust and clinically meaningful insights.

Given that IA injections are often administered to delay or avoid surgery in patients with OA, the present data indicate that readministration of PN is effective in prolonging the time to surgery, potentially reducing the need for surgery. Similar findings have been reported for HA, in which an increased injection frequency was associated with a longer duration before surgical intervention [19]. Since PN provides physical restoration through its gel-forming properties, which reduce mechanical friction [7,8,9], these findings are biologically plausible. Compared with high-molecular-weight DNA fragments extracted from salmon testes and sperm, PN exhibits a high water-binding capacity, which enhances IA viscoelasticity and contributes to physical restoration and shock absorption within the joint [20].

Thus, patients who received continuous PN injections experienced a longer delay before surgical intervention, potentially indicating a prolonged therapeutic effect. Similar patterns have been observed in previous studies on HA injections. In a systematic review, IA HA regimens with five or more injections showed statistically significant improvements in knee pain at six months [21].

Pain-related hospital visits and arthrocentesis procedure counts were evaluated as secondary indicators of disease progression and symptom management, respectively. These parameters decreased over time in both groups. The decline in pain-related hospital visits was more pronounced in the single-cycle group, suggesting a strong initial response to the PN treatment. The differing trajectories in pain-related visits between the two groups may be partly explained by underlying differences in clinical characteristics and follow-up patterns. Patients who received only a single PN cycle likely had a lower baseline symptom burden, which may lead to a more pronounced early reduction in pain-related encounters. In contrast, patients who required multiple PN cycles represent a subgroup with more persistent or recurrent symptoms and inherently greater clinical need, resulting in closer monitoring and more frequent follow-up visits. These interpretations support the notion that the observed trends may reflect differences in patient characteristics and clinical pathways rather than differences in the intrinsic effectiveness of the injections themselves. Meanwhile, the reduction rate of arthrocentesis procedures did not differ significantly between the groups, indicating that repeated PN injections maintained consistent disease-modifying effects. The decrease in arthrocentesis procedures is particularly relevant, given that prior research indicates that joint effusion and synovitis are predictive of cartilage loss and increased symptom severity. Effusion synovitis is an independent risk factor of future cartilage degeneration [22]. Moreover, previous studies have demonstrated that suprapatellar joint effusion significantly increases the risk of knee pain in patients with OA, further supporting the clinical relevance of these findings [23].

Although re-administration of PN injections showed a significant trend toward reduced NSAID use for up to six months, this effect was not observed between six and 12 months, and neither single-cycle nor re-administration of PN injections demonstrated favorable outcomes in terms of reducing antidepressant prescriptions. However, the interpretation of NSAID and antidepressant prescription rates should be approached with caution. In the Korean clinical context, with high medical accessibility, NSAIDs are often prescribed during outpatient visits, irrespective of the objective disease severity or underlying medical conditions. Previous studies conducted in Korea demonstrated that NSAIDs are frequently prescribed together with gastrointestinal medications, even in the absence of clinical necessity [24,25]. Previous reviews have suggested that NSAIDs are often prescribed regardless of the objective inflammatory activity, reflecting clinical practice patterns that rely heavily on patients’ subjective symptom reports rather than on the underlying pathophysiology [26]. Similarly, previous reviews have revealed that antidepressant prescriptions may not accurately reflect pain severity or control because they are frequently influenced by non-clinical factors, such as adherence issues and misalignment with diagnostic criteria [27,28]. Thus, NSAID and antidepressant prescription patterns may not directly reflect pain levels, and time-dependent increases or non-significant decreases may not necessarily indicate a lack of therapeutic benefit from PN. Nevertheless, these indicators were included to provide a broader perspective on healthcare utilization as a proxy for the symptom burden.

This study has several limitations. First, as a retrospective observational analysis, it remains subject to potential confounding and selection biases despite adjustments for covariates. Patients who received repeated PN injections may have had different baseline characteristics, treatment preferences, or clinical severity compared to those who received a single cycle. Although adjustments were made for covariates such as age and sex, other unmeasured factors could have influenced treatment decisions and outcomes, limiting causal inference. Importantly, the absence of radiographic severity measures, such as K-L grades, makes it difficult to determine whether differences between groups are attributable to the underlying disease severity. This limitation restricts the interpretation of time-to-surgery differences and the pace of clinical responses between the groups. Without structural severity measures, it was difficult to discern whether the patients in the repeated-cycle group progressed more rapidly, were more symptomatic at baseline, or had different treatment thresholds.

Second, the observed group differences in age, pain-related visits, and NSAID use suggest the potential for confounding factors. Patients who received repeated PN cycles may have been more symptomatic or may have had physician-driven preferences for additional injections, which could have biased the comparisons between the one- and two-cycle groups. Therefore, the results should be interpreted as associative rather than causal. However, prescription counts may not directly reflect the clinical status or pain severity. In Korea’s healthcare environment, with its high accessibility, low outpatient costs, and frequent prescribing patterns, administrative indicators (e.g., prescription frequency and visit counts) may diverge substantially from true clinical measures. Furthermore, adherence to NSAIDs and antidepressants could not be assessed because the claims data reflected prescriptions rather than actual intake. Consequently, prescription counts may not accurately represent the patients’ actual medication use, and this limitation should be considered when interpreting these indicators. Thus, NSAID or antidepressant use should not be overinterpreted as a direct surrogate of symptom intensity.

Third, this study used data from a single national health system, which limited the generalizability of the findings. Korea’s unique healthcare environment is characterized by universal insurance coverage, high outpatient accessibility, and low procedural costs [18]. These features, combined with the specific reimbursement criteria for PN, may lead to treatment patterns that differ from those in other countries. Therefore, the generalizability of these findings to other healthcare systems is limited. Therefore, these findings should be extrapolated to other countries with caution.

Finally, safety outcomes such as local or systemic adverse reactions could not be assessed directly because the claims database does not capture procedure-specific adverse events. Although previous studies demonstrated the safety and effectiveness of repeat PN treatment in patients with knee OA [11,12], structured prospective studies are needed to establish the long-term safety profile of repeat PN injections.

From a practical perspective, our findings suggest that PN re-administration may be considered for patients who experience persistent symptoms after an initial treatment period, particularly within the Korean healthcare setting where PN use follows clearly defined reimbursement criteria and is frequently applied as a conservative treatment option. However, treatment thresholds, availability of PN, and clinical decision-making frameworks differ substantially across countries. Therefore, PN re-administration should be individualized and interpreted in accordance with local practice guidelines, and further prospective international studies are needed before broader recommendations can be established.

## 5. Conclusions

This large-scale nationwide retrospective cohort study demonstrated that repeated IA PN injections are associated with sustained clinical benefits and a delayed need for surgical intervention in patients with knee OA. Compared with patients who received a single injection cycle, those who underwent multiple PN cycles showed significantly lower rates and longer times to total and partial knee arthroplasty.

In addition to improved surgical outcomes, reductions in pain-related hospital visits and arthrocentesis procedures were also observed, supporting the role of PN in symptomatic management. Although the prescription patterns for NSAIDs and antidepressants should be interpreted cautiously owing to contextual factors in clinical practice, they provide complementary insights into healthcare utilization. Importantly, patients in the readministration group were generally older and likely had more advanced disease at baseline; however, they still experienced favorable outcomes, such as lower surgical rates and longer time to surgery. However, these findings should not be interpreted as evidence of structural modifications or long-term disease-modifying effects because radiographic progression and validated patient-reported outcomes were unavailable.

In current clinical practice in Korea, intra-articular PN is typically indicated for patients with symptomatic knee osteoarthritis who remain symptomatic despite conservative management. According to the Korean Ministry of Health and Welfare reimbursement criteria (Notice No. 2021-141), intra-articular PN is reimbursed for patients with symptomatic knee osteoarthritis with Kellgren–Lawrence grade 1–3 who remain symptomatic despite conservative management [13]. This study’s results suggest that PN may be re-administered as a clinically useful nonsurgical option for delaying surgical intervention and managing symptoms in routine practice. Further studies incorporating radiographic severity measures, structural progression markers, and patient-reported outcomes are required to clarify whether the benefits of repeated PN injections extend beyond symptomatic improvement and surgical delay.

## Figures and Tables

**Table 1:** Classification of Patients Based on Sodium Polynucleotide Administration Cycles.

	1 Cycle	2 Cycles	3 Cycles	4 Cycles	5 Cycles	Number of Patients
Group 1 (single-cycle group)	O	X	X	X	X	115,388
Group 2 (Re-administration group)	O	O	X	X	X	18,453
	O	X	O	X	X	5313
	O	X	X	O	X	2315
	O	X	X	X	O	853
Total						142,322

Abbreviations: O, injection performed; X, no injection.

**Table 2:** Baseline characteristics of Study Participants.

	Total	Group 1	Group 2	*p*-Value
Number	142,322	115,388	26,934	
Age (years)	62.56 ± 10.58	62.45 ± 10.79	63.07 ± 9.65	<0.0001
Sex, male (%)	43,650 (30.67)	35,632 (30.88)	8018 (29.77)	0.0004
Number of Medical Aid beneficiaries (%)	4032 (2.83)	3259 (2.82)	773 (2.87)	0.6847
Number of outpatient visits to pain-related specialties	3.3 ± 6.04	3.25 ± 6.04	3.5 ± 6.04	<0.0001
Number of joint aspirations performed	0.23 ± 0.98	0.23 ± 0.97	0.25 ± 1.03	0.0034
Number of NSAID prescriptions	3.34 ± 4.08	3.29 ± 4.07	3.54 ± 4.09	<0.0001
Number of anti-depressant Prescriptions	0.55 ±1.92	0.54 ± 1.92	0.59 ± 1.96	0.0002

Characteristics measured during the 12-month period before the index date. Pain-related outpatient visits included visits to the rehabilitation, orthopedic surgery, anesthesiology, and neurosurgery departments. Medical Aid refers to publicly funded health coverage for low-income individuals. All values are presented as mean ± SD or number (%).

## Data Availability

The data supporting the findings of this study are available from the Korean Health Insurance Review and Assessment Service (HIRA). Restrictions apply to the availability of the data used under a license for this study. Data are available from HIRA upon reasonable request and with permission.

## Supplementary Materials

The following supporting information can be downloaded from https://www.mdpi.com/article/10.3390/jcm14238358/s1, Table S1: Surgical outcomes within 1 year after PN initiation across all groups; Table S2: Changes in pain-related hospital visits over time; Table S3: Changes in arthrocentesis procedures over time; Table S4: Changes in NSAID prescriptions over time; Table S5: Changes in depression medication prescriptions over time; Table S6: Comparison of clinical outcomes between single-cycle and re-administration groups.

## Abbreviations

The following abbreviations are used in this manuscript:

OA	Osteoarthritis
PN	Sodium Polynucleotide
IA	Intra-articular
AAOS	American Academy of Orthopaedic Surgeons
ACR/AF	American College of Rheumatology/Arthritis Foundation
K-L	Kellgren–Lawrence
NSAID	Non-steroidal Anti-inflammatory Drug
IRB	Institutional Review Board
VAS	Visual Analog Scale
PNHA	Polynucleotide plus Hyaluronic Acid
CI	Confidence Interval
SD	Standard Deviation
