# Supplementary material for: Clinical Outcomes of Repeated Sodium Polynucleotide Injections in Knee Osteoarthritis: Large-Scale, Retrospective Cohort Study
Source: J Clin Med. 2025 Nov 25;14(23):8358. doi: 10.3390/jcm14238358 (PMC12693588; doi:10.3390/jcm14238358)
Supplement: Supplementary file 1 [file jcm-14-08358-s001.zip › jcm-3990322-supplementary.pdf]

Surgery Type	Group 1<br>115388	Group 2<br>26934	P-value
**Replacement Arthroplasty of the knee**			
n (%)	5682 (4.92)	622 (2.31)	<.0001
Duration, day	176.56 (173.63 - 179.48)	252 (244.43 - 259.56)	<.0001
**Hemiarthroplasty of the knee**			
n (%)	630 (0.55)	75 (0.28)	<.0001
Duration, day	162.19 (153.72 - 170.66)	240.74 (218.92 - 262.55)	<.0001
**Osteochondral Autograft Transplantation**			
n (%)	546 (0.47)	167 (0.62)	0.0021
Duration, day	132.99 (124.49 - 141.49)	143.22 (128.43 - 158.02)	0.2266
**Autologous Chondrocyte Transplantation**			
n (%)	12 (0.01)	1 (0)	0.3012
Duration, day	190.39 (111.35 - 269.43)	-	-

Supplementary Table S1. Surgical outcomes within 1 year after PN initiation across all groups Surgery rates and timing (mean days to event) were compared between the single-cycle (Group 1) and re-administration (Group 2) groups. All the models were adjusted for age and sex. Values presented as mean (95% CI) and % of patients undergoing each procedure.

pain-related hospital visits	Comparison	Mean	Mean difference	P-value
Single-cycle group (Group 1)	Pre-treatment vs.	3.25 ± 6.05	1 (ref.)	<.0001
	6 Months Post-treatment	2.98 ± 6.3	-0.2787 (-0.3158, -0.2416)	
	6 Months Post-treatment vs.	2.98 ± 6.3	1 (ref.)	<.0001
	6 Months to 1 Year Post-treatment	2.15 ± 5.41	-0.8274 (-0.8595, -0.7953)	
Multi-cycle group (Group 2)	Pre-treatment vs.	3.51 ± 6.04	1 (ref.)	<.0001
	6 Months Post-treatment	3.08 ± 6.41	-0.4286 (-0.5115, -0.3456)	
	6 Months Post-treatment vs.	3.08 ± 6.41	1 (ref.)	<.0001
	6 Months to 1 Year Post-treatment	2.62 ± 5.96	-0.4545 (-0.5225, -0.3865)	

Supplementary Table S2. Changes in pain-related hospital visits over time. Comparisons were made between pre-treatment and 6 months post-treatment, and subsequently between 6 months and 1 year post-treatment, to evaluate the temporal trends within each group. Values are presented as mean ± SD with mean differences (95% CI).

Arthrocentesis procedures	Comparison	Mean	Mean difference	P-value
Single cycle group (Group 1)	Pre-treatment vs.	0.23 ± 0.97	1 (ref.)	<.0001
	6 Months Post-treatment	0.18 ± 0.9	-0.0533 (-0.0594, -0.0473)	
	6 Months Post-treatment vs.	0.18 ± 0.9	1 (ref.)	<.0001
	6 Months to 1 Year Post-treatment	0.11 ± 0.72	-0.062 (-0.0669, -0.0572)	
Multi-cycle group	Pre-treatment vs.	0.25 ± 1.03	1 (ref.)	<.0001

(Group 2)				
	6 Months Post-treatment	0.18 ± 0.9	-0.0701 (-0.0842, -0.056)	
	6 Months Post-treatment vs.	0.18 ± 0.9	1 (ref.)	<.0001
	6 Months to 1 Year Post-treatment	0.13 ± 0.77	-0.0467 (-0.057, -0.0364)	

Supplementary Table S3. Changes in Arthrocentesis procedures over time. Comparisons were made between pre-treatment and 6 months post-treatment, and subsequently between 6 months and 1 year post-treatment, to evaluate the temporal trends within each group. Values are presented as mean ± SD with mean differences (95% CI).

NSAID prescriptions	Comparison	Mean	Mean difference	P-value
Single cycle group (Group1)	Pre-treatment vs.	3.29 ± 4.07	1 (ref.)	<.0001
	6 Months Post-treatment	3 ± 3.89	-0.2886 (-0.3112, -0.2661)	
	6 Months Post-treatment vs.	3 ± 3.89	1 (ref.)	0.0004
	6 Months to 1 Year Post-treatment	2.97 ± 3.77	-0.0372 (-0.0577, -0.0167)	
Multi-cycle group (Croup2)	Pre-treatment vs.	3.54 ± 4.09	1 (ref.)	<.0001
	6 Months Post-treatment	3.44 ± 3.81	-0.1011 (-0.1513, -0.0508)	
	6 Months Post-treatment vs.	3.44 ± 3.81	1 (ref.)	0.8151
	6 Months to 1 Year Post-treatment	3.44 ± 3.78	0.005 (-0.037, 0.047)	

Supplementary Table S4. Changes in NSAID prescriptions over time. Comparisons were made between pre-treatment and 6 months post-treatment, and subsequently between 6 months and 1 year post-treatment, to evaluate the temporal trends within each group. Values are presented as mean ± SD with mean differences (95% CI).

Depression medication prescriptions	Comparison	Mean	Mean difference	P-value
Single cycle group (Group 1)	Pre-treatment vs.	0.54 ± 1.92	1 (ref.)	<.0001

	6 Months Post-treatment	0.56 ± 1.91	0.0217 (0.0137, 0.0297)	
	6 Months Post-treatment vs.	0.56 ± 1.91	1 (ref.)	0.7547
	6 Months to 1 Year Post-treatment	0.56 ± 1.91	0.0012 (-0.0062, 0.0086)	
Multi-cycle group (Group 2)	Pre-treatment vs.	0.59 ± 1.96	1 (ref.)	0.002
	6 Months Post-treatment	0.62 ± 1.95	0.0323 (0.0118, 0.0527)	
	6 Months Post-treatment vs.	0.62 ± 1.95	1 (ref.)	0.1469
	6 Months to 1 Year Post-treatment	0.61 ± 1.92	-0.0116 (-0.0272, 0.0041)	

Supplementary Table S5 Changes in depression medication prescriptions over time. Comparisons were made between pre-treatment and 6 months post-treatment, and subsequently between 6 months and 1 year post-treatment, to evaluate the temporal trends within each group. Values are presented as mean ± SD with mean differences (95% CI).

Supplementary Table S2~S5. To compare the rate of change in clinical outcomes across the groups, ANCOVA models adjusted for age and sex were used. The outcomes included hospital visits to pain-related specialties, arthrocentesis procedures, NSAID prescriptions, and antidepressant prescriptions. A paired T-test was used to determine temporal changes within each group.

Number	Single cycle group (Group 1) 115388			Multi-cycle group (Group 2) 26934			P-value
	6 months Pre-treatment vs.	6 Months Post-treatment	6 Months to 1 Year Post-treatment	6 months Pre-treatment vs.	6 months Post-treatment	6 months to 1 Year Post-treatment	
**Pain-related hospital visits**							
Mean							
Unadjusted	3.25 ± 6.05	2.98 ± 6.3	2.15 ± 5.41	3.51 ± 6.04	3.08 ± 6.41	2.62 ± 5.96	<.0001
Age, sex Adjusted	3.2 (3.17, 3.24)	2.9 (2.86, 2.94)	2.07 (2.04, 2.11)	3.41 (3.33, 3.48)	2.95 (2.88, 3.03)	2.51 (2.44, 2.57)	<.0001
**Arthrocentesis procedures**							
Mean							
Unadjusted	0.23 ± 0.97	0.18 ± 0.9	0.11 ± 0.72	0.25 ± 1.03	0.18 ± 0.9	0.13 ± 0.77	0.7029
Age, sex Adjusted	0.25 (0.24, 0.25)	0.19 (0.18, 0.19)	0.12 (0.12, 0.13)	0.27 (0.25, 0.28)	0.19 (0.18, 0.2)	0.14 (0.13, 0.15)	0.7023
**NSAIDs prescriptions**							
Mean							
Unadjusted	3.29 ± 4.07	3 ± 3.89	2.97 ± 3.77	3.54 ± 4.09	3.44 ± 3.81	3.44 ± 3.78	<.0001
Age, sex Adjusted	3.29 (3.26, 3.31)	2.98 (2.96, 3.01)	2.94 (2.92, 2.96)	3.51 (3.47, 3.56)	3.39 (3.35, 3.44)	3.39 (3.35, 3.44)	<.0001
**Depression medication prescriptions**							
Mean							
Unadjusted	0.54 ± 1.92	0.56 ± 1.91	0.56 ± 1.91	0.59 ± 1.96	0.62 ± 1.95	0.61 ± 1.92	0.7453
Age, sex Adjusted	0.51 (0.5, 0.53)	0.53 (0.52, 0.54)	0.54 (0.52, 0.55)	0.56 (0.54, 0.58)	0.59 (0.56, 0.61)	0.58 (0.55, 0.6)	0.7321

Supplementary Table S6. Comparison of clinical outcomes between the single- and multi-cycle groups. Mean values (± standard deviation) are presented for each time point (6 months pre-treatment, 6 months post-treatment, and 6 months to 1 year post-treatment). The outcomes included NSAID prescriptions, arthrocentesis procedures, pain-related hospital visits, and depression medication prescriptions. Age- and sex-adjusted values are also provided. Values represent mean ± SD and age- and sex-

adjusted estimates (95% CI). P-values represent differences in the slopes estimated from the linear regression models between the two groups.
